# Involvement of the Neutral Amino Acid Transporter SLC6A15 and Leucine in Obesity-Related Phenotypes

**DOI:** 10.1371/journal.pone.0068245

**Published:** 2013-09-04

**Authors:** Jana Drgonova, Josefin A. Jacobsson, Joan C. Han, Jack A. Yanovski, Robert Fredriksson, Claude Marcus, Helgi B. Schiöth, George R. Uhl

**Affiliations:** 1 Molecular Neurobiology Branch, NIH-IRP, NIDA, Baltimore, Maryland, United States of America; 2 Unit of Functional Pharmacology, Department of Neuroscience, Uppsala University, Uppsala, Sweden; 3 Section on Growth and Obesity, Program in Developmental Endocrinology and Genetics, NICHD-IRP, NIH 10 CRC, Bethesda, Maryland, United States of America; 4 Division of Pediatrics, Department of Clinical Science, Intervention and Technology, Karolinska Institutet, Stockholm, Sweden; CRCHUM-Montreal Diabetes Research Center, Canada

## Abstract

Brain pathways, including those in hypothalamus and nucleus of the solitary tract, influence food intake, nutrient preferences, metabolism and development of obesity in ways that often differ between males and females. Branched chain amino acids, including leucine, can suppress food intake, alter metabolism and change vulnerability to obesity. The *SLC6A15* (v7-3) gene encodes a sodium-dependent transporter of leucine and other branched chain amino acids that is expressed by neurons in hypothalamus and nucleus of the solitary tract. We now report that *SLC6A15* knockout attenuates leucine's abilities to reduce both: a) intake of normal chow and b) weight gain produced by access to a high fat diet in gender-selective fashions. We identify SNPs in the human *SLC6A15* that are associated with body mass index and insulin resistance in males. These observations in mice and humans support a novel, gender-selective role for brain amino acid compartmentalization mediated by *SLC6A15* in diet and obesity-associated phenotypes.

## Introduction

Amino acids can influence weight control by mechanisms that include enhanced postprandial and post absorptive satiety [1], [2], energy expenditure [3], [4] and insulin action [5], [6]. Hypothalamic neurons sense amino acid concentrations and integrate this data with information from other signals [7], [8]. Interest in the ways in which amino acids influence nutrition and body weight is boosted by increasing rates of obesity [9], [10] and elucidation of some of the genetic influences on these processes [11], [12], [13], [14], [15].

Essential branched-chain amino acids (BCAA), including leucine, constitute up to 20% of dietary protein and make significant contributions to the influences of protein on body weight [16]. Hypothalamic concentrations of BCAA rise rapidly after protein ingestion [17], [18]. Dietary administration of leucine or mixtures of BCAA increase thermogenesis, augment protein synthesis and alter insulin actions [6], [19], [20]. Leucine administered into the brain decreases food intake and body weight while increasing hypothalamic signaling through mTOR pathways [21], [22]. Firing rates of key arcuate/mediobasal hypothalamic neurons increase promptly upon application of leucine in ways that correspond to increased cFos expression in these hypothalamic neurons and interconnected neurons of the nucleus of the solitary tract [22].

SLC6A15, a founding member of a subfamily of “orphan neurotransmitter transporters” [23], [24], [25], mediates sodium-dependent, electrogenic transport of leucine and other BCAA [26], [27]. SLC6A15 is expressed in plasma membranes of neurons [26] in brain areas that include the arcuate/mediobasal hypothalamus and the nucleus of the solitary tract [28], [29]. SLC6A15 is thus well positioned to regulate cellular compartmentalization of leucine and other BCAA in ways that could contribute to the influences of these nutrients on food intake, body weight and other obesity-related phenotypes. Reported associations between human obesity and genomic markers in a related neutral amino acid transporter, SLC6A14, support the idea that human variations in other neutral amino acid transporters might also play significant roles in obesity-related traits [30], [31], [32]. There is also some evidence for gender influences; SLC6A14 variants exert sex-selective effects on fat oxidation, for example [32].

We have recently created SLC6A15 knockout mice that display 40% reductions in sodium-dependent leucine uptake into brain synaptosomes, lack gross disabilities and present few behavioral differences from wild-type littermates [33]. We now report use of these knockout mice to assess roles for SLC6A15 in regulation of food intake and body weight by dietary leucine in male and female mice fed a high fat diet. We also report testing associations between SLC6A15 genomic markers with obesity-related phenotypes in two independent human samples. The findings support roles for SL6A15 on diet and obesity that are more prominent in males.

## Subjects and Methods

### Mice

SLC6A15 knockout (KO) mice lack functional SLC6A15 transporter due to deletion of exon three, which codes for the transporter's first transmembrane domain, as previously described [33]. Congenic SLC6A15 mice used in the present study were created by back-crossing the original C57BL/6J-129S6/SvEvTac mixed background mice with C57BL/6J mice for 10 generations. Mice were maintained at 24°C with a 12∶12-h light/dark cycle. Offspring produced by heterozygous breeding were genotyped by polymerase chain reaction (PCR) of DNA from ear biopsy samples as described previously [33]. At age 8–9 weeks, homozygote KO and wild-type (WT) mice with *ad libitum* access to water and rodent chow (Teklad Global 2020X Diet, Harlan Laboratories, Madison, WI) containing 16% calories from fat, and 24% calories from protein, were housed individually in standard shoebox cages that were enriched with nestlets and igloos. Food and water intake was monitored daily and animals were weighed once/week. Consumption was expressed as kcal per kg body weight per day based on the weight at the beginning of the given week. All animal experiments were approved by the National Institute on Drug Abuse Animal Care and Use Committee and conducted in accordance with the National Institute of Health Guide for the Care and Use of Laboratory Animals (NIH Publications No. 80-23) revised 1996.

### Human Subjects

#### Swedish research volunteers

932 subjects included: a) 222 girls and 198 boys (age 2–20 years; body mass index (BMI) standard deviation score (BMI SDS) 6.2±1.4) enrolled at the National Childhood Obesity Centre, Karolinska University Hospital, Huddinge, Sweden and b) 263 healthy girls and 249 healthy boys (15–20 years; BMI SDS 0.2±0.9) recruited from 17 upper secondary schools around Stockholm. The study was approved by the Regional Committee of Ethics, Stockholm. Written consent was obtained from all the participants, or, in case of minors, from their guardians. Body weight and height were measured. BMI was calculated as body weight (kg) divided by squared height (m). BMI SDS was calculated from weight, height, age and sex based on [34].

#### NIH research volunteers

362 girls and 293 boys, age 2–18 years, 37.4% African American, 55.3% non-Hispanic Caucasian, were recruited for metabolic protocols. The cohort was enriched for obese participants (BMI ≥95th percentile; clinicaltrials.gov: NCT00320177, NCT00001195, and NCT00001522). Studies were described to children and their parents and written informed assent from children and written informed consent from parents were obtained. Study protocols were approved by the Eunice Kennedy Shriver National Institute of Child Health and Human Development Institutional Review Board. Each participant's weight and height were measured. BMI SD scores (BMI z scores) were calculated according to Centers for Disease Control and Prevention 2000 growth charts which account for the stages of pubertal development [35].

## Methods

### Mice

#### Effect of leucine on food consumption

After one week habituation to individual cages, at age 9–10 weeks, WT males weighed 22.62±1.68 g (standard deviation, SD), KO males 22.24±1.77 g, WT females 17.98±1.05 g, and KO females weighed 18.09±1.69 g. Baseline food and water intakes were monitored daily for four consecutive days. During the following week, drinking water was supplemented with leucine (1.5%, Sigma, St. Louis, MO) as described by Nairizi and Zhang [36], [37] and daily food and fluid intakes were again monitored for four consecutive days.

#### Two-bottle leucine preference test

Naive 9–10 week old mice weighing 23.55±2.02 g SD (WT males), 22.69±2.76 g (KO males), 18.25±1.28 g (WT females), and 18.14±1.42 g (KO females), were submitted to two-bottle choice testing. For 12 days, animals in their home cages were offered access to chow and two bottles, one filled with water and one containing 1.5% leucine solution. The positions of the bottles were switched daily. Average daily intake was calculated from the last nine days of the experiment.

#### Effect of lysine on food consumption

This experiment was carried out with a new group of naive 9–10 week old mice. WT males weighed 22.03±1.88 g, KO males 22.15±1.55 g, WT females 17.44±1.03 g, and KO females weighed 18.01±2.09 g. Lysine (Sigma) was supplemented as 1.8% solution, a concentration equicaloric with the leucine solution [38].

#### Effects of leucine on high fat diet-induced obesity

A new cohort of 15 week-old mice weighing 26.90±1.94 g SD (WT males), 25.80±2.21 g (KO males), 20.52±1.34 g (WT females), 21.27±1.40 g (KO females) was supplemented with high fat diet (45% calories from fat, 19% calories from protein; TD.06415, Harlan Laboratories, Frederick, MD) and 1.5% leucine in drinking water for 9 weeks. A control group was fed the same high fat diet but without the supplemental leucine: 25.98±2.14 g SD (WT males), 25.56±1.79 g (KO males), 20.02±1.79 g (WT females), 20.73±1.68 g (KO females). Food and water consumption were monitored three times per week and animals were weighed once per week.

#### Abdominal Fat, Serum and genotype analysis

Animals were euthanized by decapitation; trunk blood was collected and allowed to clot for 10 min at room temperature. Abdominal fat pads were dissected and weighed. Serum was obtained by centrifugation at 1 000×RCF for 10 min and stored at −80°C until use. Serum glucose was measured with the Glucometer Elite (Bayer), serum insulin was determined by radioimmunoassay (Millipore, St. Charles, MO), and serum triglycerides with a colorimetric assay 2780-400H (Thermo Electron, Louisville, CO,).

#### RT PCR

Animals with *ad libitum* access to water and rodent chow (Teklad Global 2020X Diet) were euthanized by decapitation, brains were removed rapidly, hypothalami were dissected on an ice-cold plate, and mRNA was isolated with Qiagen RNeasy lipid tissue mini kit (Qiagen, Valencia, CA) according to the manufacturer's instructions. cDNA was synthesized with the ThermoScript RT-PCR system (Invitrogen, Carlsbad, CA). Levels of AGRP, CART, NPY, and POMC mRNAs were assessed by means of the relative calibration curve method with SybrGreen PCR Master Mix (Applied Biosystems, Carlsbad, California) on the ABI Prism 7900 HT equipment. Each 10 µl PCR reaction contained 5 µl PCR Master Mix, 0.2 µl cDNA, and 0.25 µM primers. Thermal cycling was carried out as follows: 95°C for 5 min and 40×95°C for 15 s, 60°C for 15 s and 72°C for 15 s. Data were collected at 72°C. Expression levels of peptides were normalized to the geometric mean of GAPDH, HPRT, and UBC mRNAs. The primers used were: qAGRPf (GTCTAAGTCTGAATGGCCTCAA) with qAGRPr (ACTCGCGGTTCTGTGGATCTA), qCARTf (GCGTTGCAAGAAGTCCTGAAG) with qCARTr (CACTGCTCTCCAGCGTCACA), qNPYf (GGCAAGAGATCCAGCCCTGA) with qNPYr (CCACATGGAAGGGTCTTCAAG), qPOMCf (ATGTGTGGAGCTGGTGCCTG) with qAGRPr (AGCGAGAGGTCGAGTTTGCAA), qGAPDHf (GCATGGCCTTCCGTGTTC) with qGAPDHr (CACCACCTTCTTGATGTCATC), qHPRTf (GACACTGGTAAAACAATGCAAAC) with q HPRTr (GAGGTCCTTTTCACCAGCAAG), and qUBCf (GCAGGCAAGCAGCTGGAAGA) with q UBCr (TTCACAAAGATCTGCATCCCAC).

### Humans

#### Homeostatic model assessment-insulin resistance (HOMA-IR)

Plasma glucose and insulin were measured from blood samples drawn after 12-h overnight fast as previously described [39]. HOMA-IR was calculated as fasting serum insulin (mU/L)×plasma glucose (mmol/L)/22.5.

#### Genotyping

Genomic DNA was extracted from blood samples using Qiagen kits (Qiagen, Hilden, Germany and QIAGEN Inc, Valencia, CA) or by a commercial company (Lofstrand Laboratories Ltd, Gaithersburg, MD). Two SNPs in the SLC6A15 promoter region, rs11116654 and rs7980296, and one within the *hSLC6A15* gene, rs17183577, were genotyped using allelic discrimination assays (Applied Biosystems part numbers ABI: c_32172710_10; ABI: c_29406847_10; ABI: c_34160772_10) and ABI Prism 7900HT Sequence Detection System according to the manufacturer's protocol, with automated genotype calling. 97.6% (rs11116654), 97.4% (rs7980296), and 99.3% (rs17183577) of SNPs in the NIH sample and 97.9% of all three SNPs in the Swedish sample passed quality control measures.

### Statistical analyses

NIH samples were analyzed by linear regression assuming an additive model with SPSS version 17.0 (IBM Corporation, Armonk, NY). Swedish samples were analyzed by linear regression assuming an additive model with PLINK, version 1.05, Shaun Purcell (http://pngu.mgh.harvard.edu/purcell/plink/) [40]. Pearson's X^2^-test was applied to both sample sets to test for deviations from Hardy-Weinberg equilibrium. Quantitative skewed variables were log-transformed before analysis. BMI-Z for the NIH samples was also adjusted for race, while HOMA-IR was adjusted for age, race, and BMI-Z for the NIH samples and for age and BMI SDS for Swedish samples. Initial testing of samples of both sexes combined was followed by testing of samples by sex, based on data from knockout mice. Differences were considered statistically significant at p<0.05.

Mouse data were expressed as mean ± SEM and assessed by repeated measures analysis of variance (ANOVA) with sex and genotype as factors (PASW Statistics 18, SPSS, Inc.). Where ANOVA showed statistical significance, Scheffe's *post hoc* comparisons were made between the treatments separately for each genotype.

## Results

### Mice

#### Effect of leucine on consumption of standard lab chow

**Figure 1:**
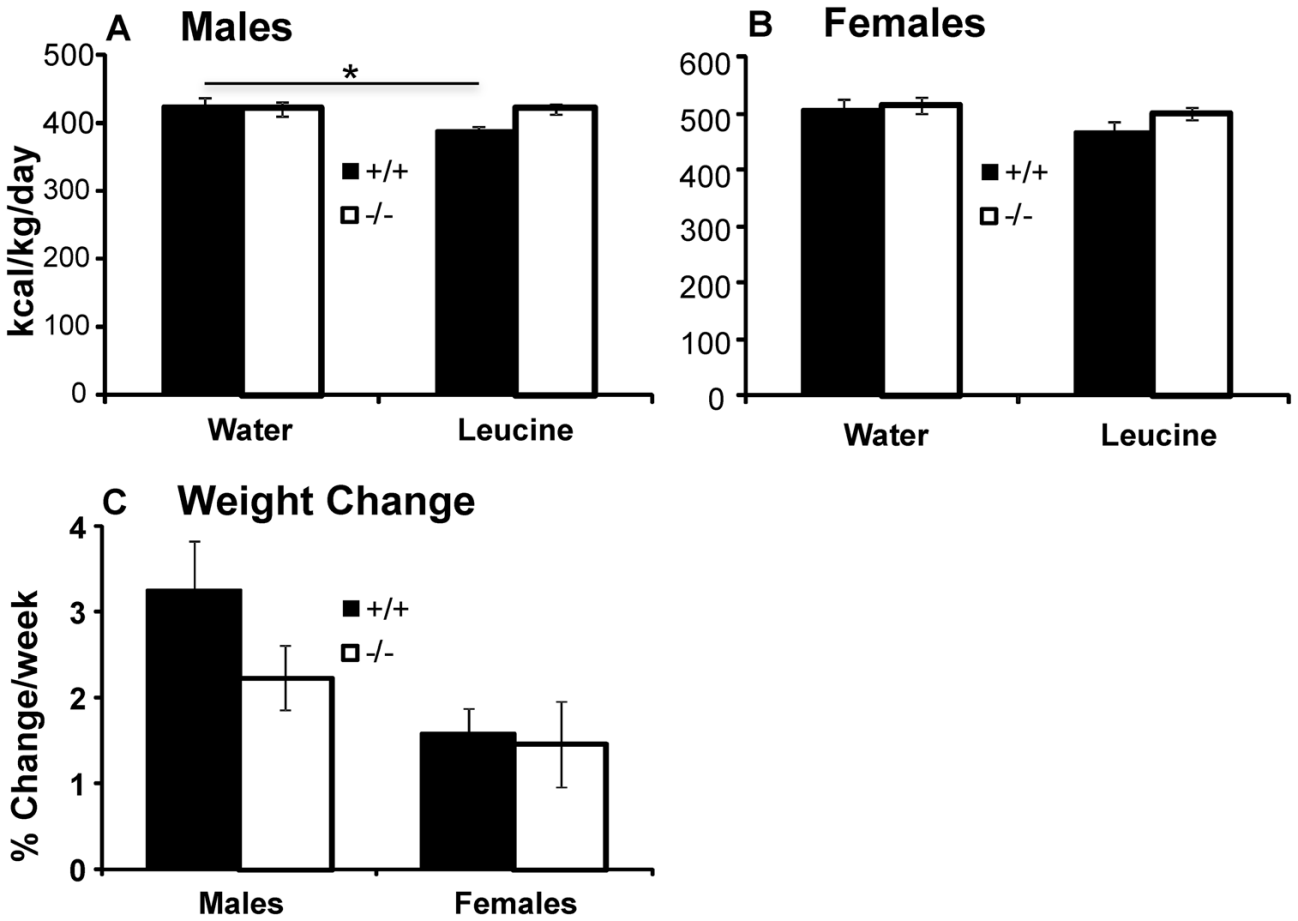
Effect of Leucine on consumption in mice. **A**. Only WT males were sensitive to the appetite-suppressing effects of leucine (ANOVA effect of leucine * genotype p = 0.040; Scheffe's *post-hoc* test p = 0.020 for WTs), while leucine had no effect on the SLC6A15 KO males (Scheffe's *post-hoc* test p = 0.871). **B**. In females, leucine had the same effect on food intake in both genotypes (ANOVA effect of leucine p = 0.063; effect of leucine * genotype p = 0.354). **C**. During leucine treatment, WT and KO mice gained weight at equal rates (t-test p = 0.141; for males; p = 0.830 for females). Data are expressed as means ± SEM; n = 13–14/group.

To evaluate the involvement of the SLC6A15 amino acid transporter in the appetite-suppressing effects of leucine, we first monitored consumption of standard lab chow for four days with and without leucine supplementation of drinking water in WT and KO mice. This experimental arrangement has been successfully used in previous studies [36], [37], [41]. Baseline calorie consumption was essentially the same in mice of both genotypes, though different between males and females (ANOVA effect of sex on calorie intake/g body weight/day; p<0. 001) (Figure 1A and B). After addition of leucine to drinking water, we observed a significant leucine * genotype interaction in males (Figure 1A; repeated measures ANOVA effect of leucine * genotype p = 0.040). Leucine markedly decreased consumption of chow in WT males (Scheffe's post-hoc p = 0.020) but not in KO males (Scheffe's post-hoc p = 0.871). The higher calorie intake in KO males did not lead to higher weight increase, however (Figure 1C; t-test effect of genotype p = 0.141). By contrast, WT and KO females displayed similar effects of leucine on calorie consumption (Figure 1B; ANOVA effect of leucine p = 0.063; leucine * genotype p = 0.354). During this experiment, we noted that both KO males and females consumed significantly more fluids; this difference was further exacerbated by leucine supplementation (Figure S1).

#### Leucine preference in two-bottle choice

**Figure 2:**
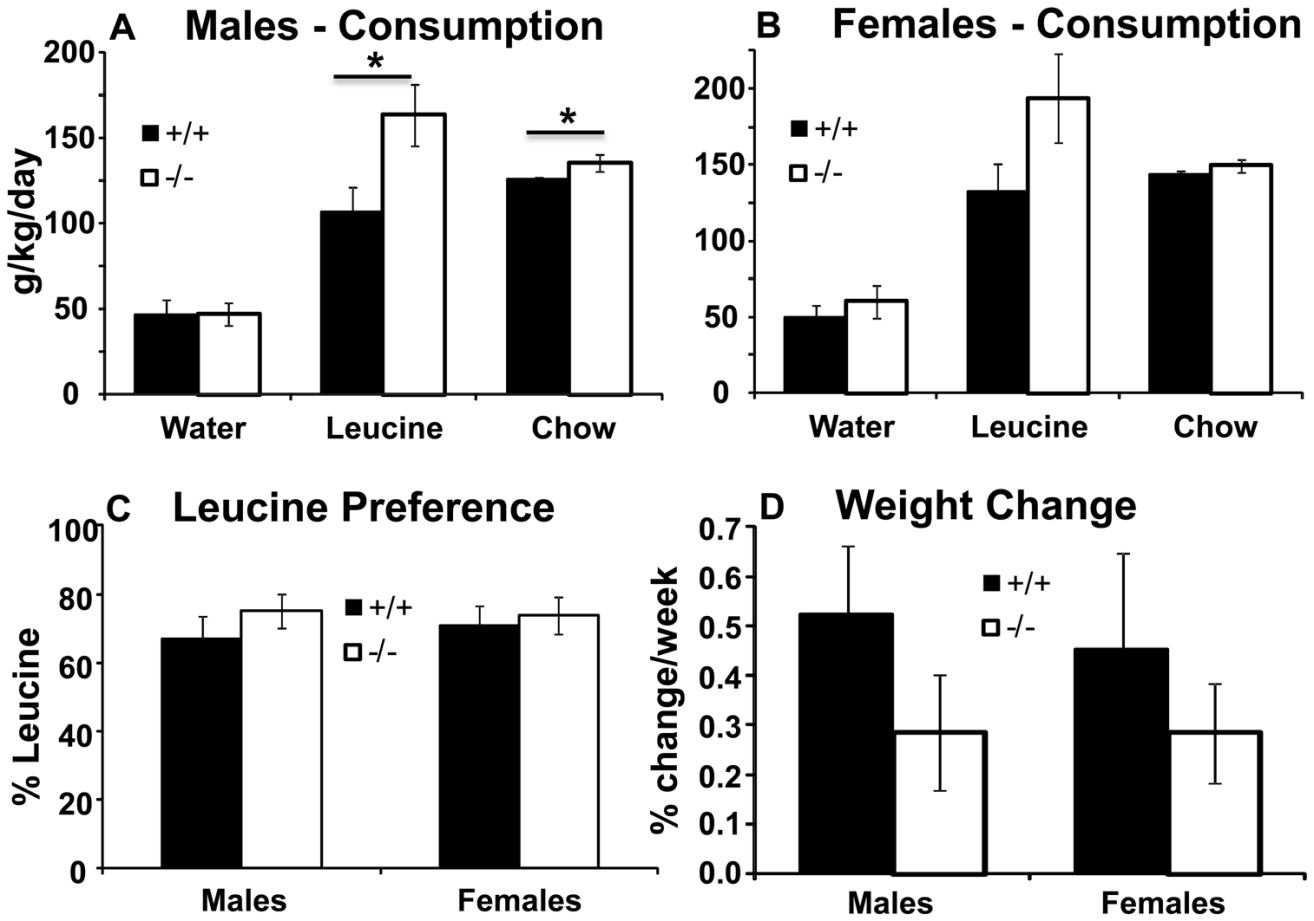
Leucine preference in SLC6A15 mice. **A**. Male SLC6A15 KO mice consumed significantly more leucine (t-test p = 0.015) and chow (t-test p = 0.046) than WT controls. **B**. Female SLC6A15 KO mice consumed a similar quantity of leucine (t-test p = 0.059) and chow (t-test p = 0.210) as WT controls. **C**. Leucine preference did not differ between the sexes and genotypes (ANOVA effect of sex p = 0.786, effect of genotype p = 0.318). **D**. During leucine preference test, WT and KO mice gained weight at equal rates (t-test p = 0.180; for males; p = 0.696 for females). Data are expressed as means ± SEM; n = 12–13/group.

Since in the previous experiment SLC6A15 KO mice consumed significantly more leucine than their WT siblings, we submitted a new group of naive animals to a two-bottle choice test to determine if SLC6A15 KO mice display preference for consumption of this amino acid. Male and female mice of both genotypes preferred to drink leucine solution (Fig. 2A and B; ANOVA p<0.001 for all). However, there were no differences in preference for leucine in WT *vs* KO mice (Fig. 2C; t-test p = 0.318 for males and 0.703 for females). As in the previous experiment, SLC6A15 KO males consumed significantly more calories than the WT males (t-test p = 0.031), while in females the difference between the two genotypes did not reach statistical significance (t-test p = 0.120). The higher calorie intake in males did not lead to a significantly greater weight increase, however (Figure 2D; t-test effect of genotype p = 0.180 in males, and p = 0.696 in females).

#### Effect of lysine on consumption of standard chow

**Figure 3:**
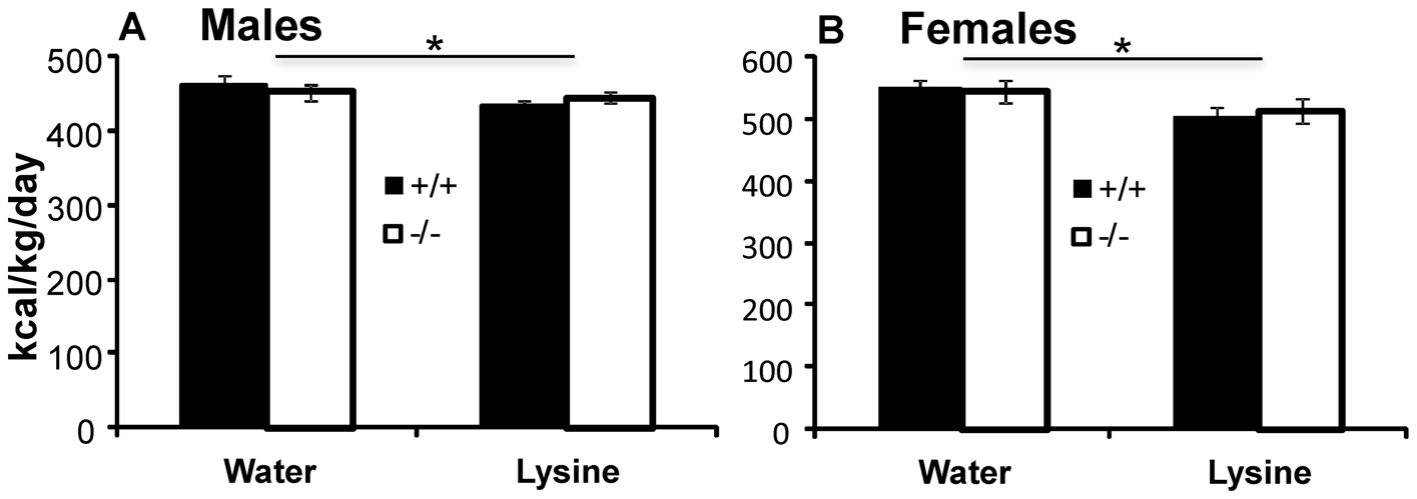
Effect of Lysine on consumption in mice. **A**. Lysine suppressed appetite in both WT and KO males (repeated measures ANOVA effect of lysine p = 0.031; lysine * genotype p = 0.154). **B**. Lysine suppressed appetite equally in WT and KO females (effect of lysine p = 0.011; lysine * genotype p = 0.596). Data are expressed as means ± SEM; n = 12/group.

To provide a control, we examined the effects of lysine on consumption of standard chow in WT and KO mice. While lysine is an essential amino acid that may share similar appetite suppressing properties with leucine [42], it is not efficiently transported by SLC6A15 [26], [27]. Preference for this amino acid did not differ between mice of different sexes or genotypes. All consumed about 50% of this amino acid solution in two-bottle preference testing (Figure S3). In single-bottle test, adding 1.8% lysine (equicaloric to leucine concentrations) to drinking water significantly suppressed appetite in both WT and KO males (Fig. 3A; repeated measures ANOVA effect of lysine p = 0.031) and females (Fig. 3B; p = 0.011). Adding lysine to drinking water did significantly increase drinking in KO males (Figure S2) and sex had a significant effect on consumption (ANOVA sex p<0.001). These data provide evidence for specificity of the knockout: there was no significant lysine * genotype interaction in either sex (p = 0.154 in males, p = 0.596 in females).

#### Expression of orexigenic and anorexigenic peptides

**Figure 4:**
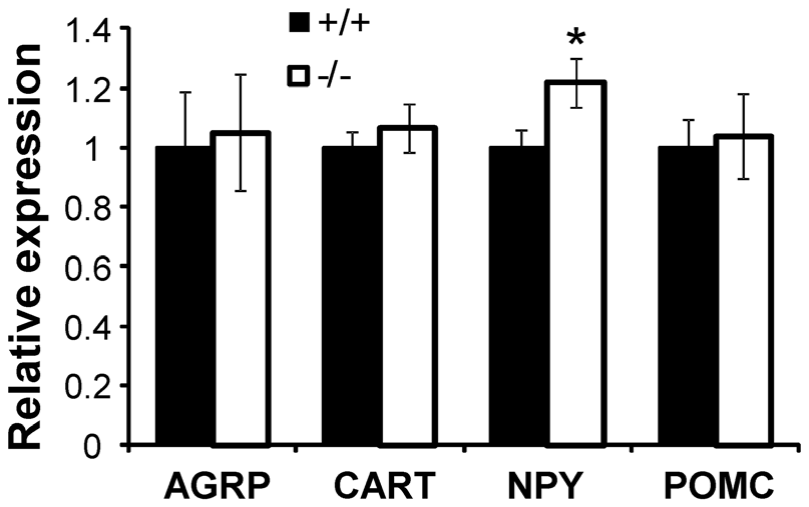
mRNA levels of orexigenic and anorexigenic peptides in the hypothalamus of SLC6A15 mice. SLC6A15 KO males with *ad libitum* access to water and rodent chow expressed significantly higher levels of the orexigenic peptide NPY in their hypothalamus (t-test p = 0.024). Data are expressed as means ± SEM; n = 11/group.

To determine if SLC6A15 deletion affects food consumption in males through altered expression of orexigenic or anorexigenic peptides, we measured their mRNA levels in the hypothalamus. While there were no differences between the WT and SLC6A15 KO males in expression levels of AGRP, CART, or POMC, (t-test p = 0.850, 0.494, and 0.821, respectively), SLC6A15 KO males expressed significantly higher levels of the orexigenic peptide NPY (Figure 4; t-test p = 0.024).

#### Effects of leucine on high fat diet

**Figure 5:**
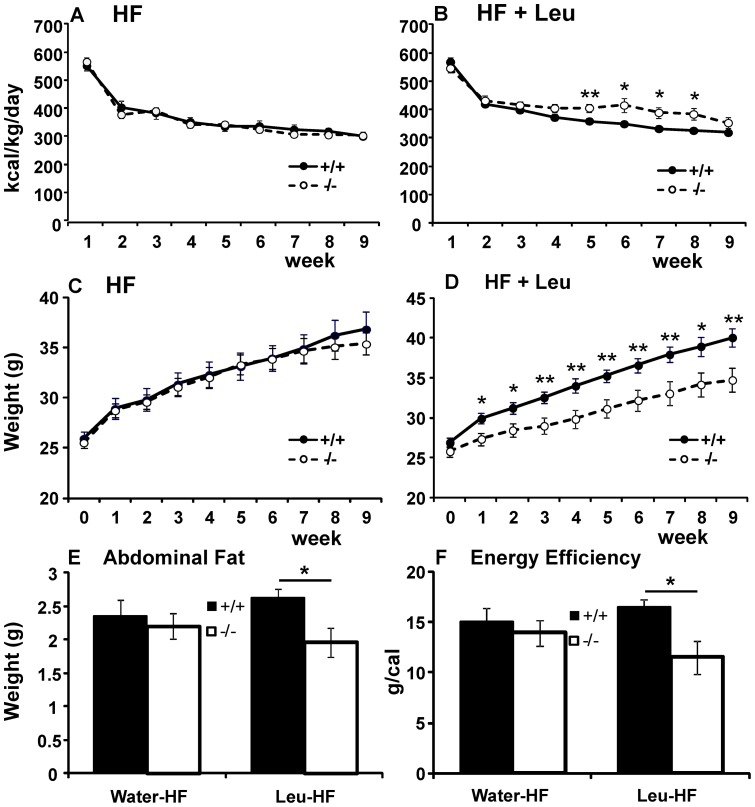
Effect of high fat diet and leucine supplementation on SLC6A15 males. **A**. KO and WT males on high fat diet consumed the same amount of calories (repeated measures ANOVA genotype * week p = 0.649) **B**. KO males consumed more calories than WT males when high fat diet was supplemented with leucine (repeated measures ANOVA genotype * week p = 0.009). **C**. KO and WT males on high fat diet gained weight at the same rate (repeated measures ANOVA genotype * week p = 0.628). **D**. KO males gained less weight than WT males when high fat diet was supplemented with leucine (repeated measures ANOVA genotype * week p = 0.014). **E**. Without leucine supplementation, there was no difference in the abdominal fat pads weights between WT and KO males (t-test p = 0.645). When supplemented with leucine, KO males accumulated less abdominal fat than their WT littermates (t-test p = 0.017). **F**. Without leucine supplementation, there was no difference in the energy efficiency, calculated as body weight gain in grams/total calorie consumption in calories, between WT and KO males (t-test p = 0.570). When supplemented with leucine, energy efficiency of KO males was significantly lower than that of WT controls (t-test p = 0.010). Data are expressed as means ± SEM; n = 12/group.

As leucine has been reported to suppress diet-induced obesity in mice [37], [43], we examined its effects on high fat diet-induced obesity in SLC6A15 KO mice. To this end, we monitored consumption of high fat chow with and without leucine supplementation in WT and KO animals. Because of the highly significant differences between males and females in consumption and weights (ANOVA effect of sex p<0. 001 for each), we evaluated the two sexes separately. In males, diet interacted significantly with genotype to affect consumption (repeated measures ANOVA diet * GT p = 0.010). On HF diet supplemented with leucine, KO males consumed more calories/kg/day than their WT littermates (Figure 5B; repeated measures ANOVA effect of GT p = 0.006). The increased consumption in KO males with leucine supplementation did not translate into increased weight gain. On the contrary, KO males gained significantly less weight on leucine-supplemented HF diet than the WT males (Figure 5D; repeated measures ANOVA p = 0.009). Thus leucine supplementation lead to significantly lower energy efficiency in the KO males compared to their WT siblings (Figure 5F; t-test p = 0.010).

Without leucine supplementation, consumption, weight gain, and energy efficiency did not differ between the genotypes (Figure 5A, C, and F repeated measures ANOVA p = 0.575 and 0.832, t-test p = 0.570, respectively). Analysis of leucine's effects in each genotype revealed that on high-fat diet, leucine exerted no significant effect on calorie intake and weight in WT males, but it did increase calorie intake without increasing the weight gain of KO males (figure S4 and S6). To shed some light on the effects of leucine supplementation on body composition, we determined weights of the abdominal fat pads at the end of the experiment. Leucine-supplemented SLC6A15 KO males accumulated significantly less abdominal fat than leucine-supplemented WT controls (Figure 5E; t-test p = 0.017), in ways that closely paralleled the differences in total weight gained (figure S6).

**Figure 6:**
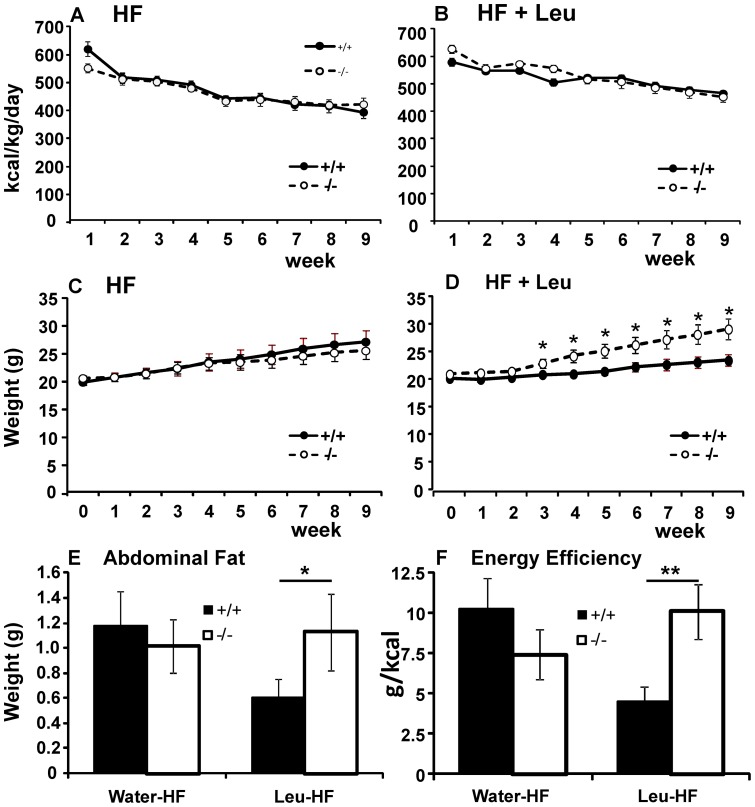
Effect of high fat diet and leucine supplementation on SLC6A15 females. **A**. and **B**. SLC6A15 KO females did not differ from WT controls in calorie intake without (**A**. repeated measures ANOVA p = 0.633) or with leucine supplementation (**B**. repeated measures ANOVA p = 0.585). **C**. KO females on HF diet gained weight at a similar rate to their WT siblings (repeated measures ANOVA p = 0.790). However, when HF diet was supplemented with leucine, KO females gained significantly more weight than WT females (repeated measures ANOVA p = 0.023). **E**. Regardless of diet, KO females did not accumulate abdominal fat differently than their WT littermates (t-test p = 0.633 for HF diet without leucine and p = 0.100 for HF with leucine supplementation). **F**. Without leucine supplementation, there was no difference in the energy efficiency, calculated as body weight gain in grams/total calorie consumption in calories, between WT and KO females (t-test p = 0.237). When supplemented with leucine, energy efficiency of KO females was significantly higher than that of WT females (t-test p = 0.007). Data are expressed as means ± SEM; n = 12/group.

When intake of calories/kg/day in females was evaluated, no significant interaction between diet and genotype was observed (Figure 6A and B; repeated measures ANOVA diet * GT p = 0.466). KO females on HF diets gained weight at a rate similar to that noted in their WT siblings (Figure 6C; repeated measures ANOVA effect of GT p = 0.790). However, when HF diet was supplemented with leucine, KO females gained significantly more weight than WT females (Figure 6D; repeated measures ANOVA effect of GT p = 0.023), despite consuming the same amount of calories/kg/day (Figure 6B; repeated measures ANOVA effect of GT p = 0.307). The increased weight gain in SLC6A15 KO females paralleled their increased energy efficiency (Figure 6F, t-test p = 0.007) but did not parallel their abdominal fat accumulation as closely. Although KO females showed trends toward increased abdominal fat pad weights, this difference did not reach statistical significance (Figure 6E, t-test p = 0.100). Analysis of leucine's effects in females of each genotype revealed that on high-fat diets, leucine increased calorie intake in both WT and KO females without significant effects on their weights (Figure S5).

#### Serum Parameters

**Figure 7:**
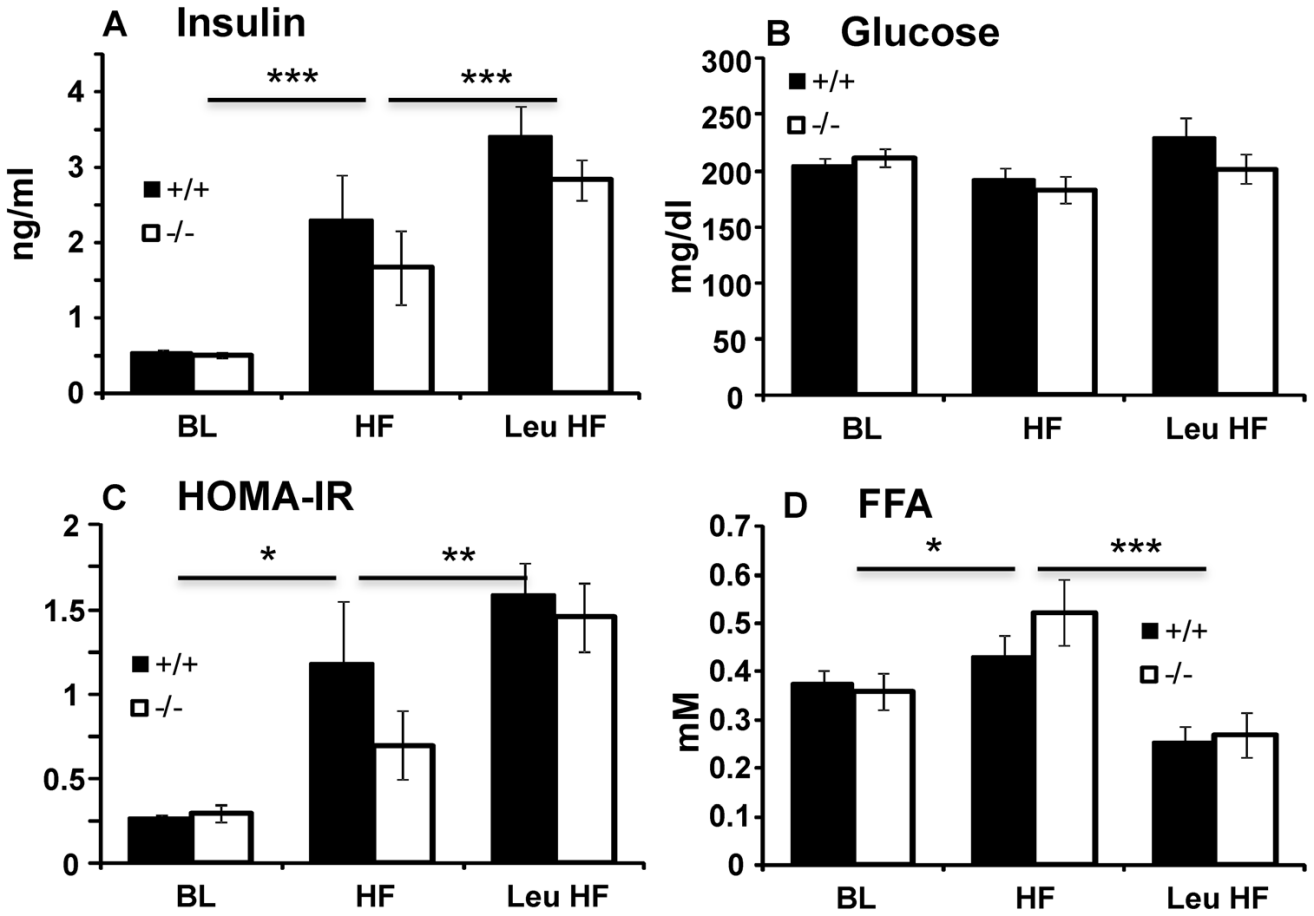
Effect of high fat diet and leucine supplementation on serum parameters in SLC6A15 males. **A**. High fat diet increased plasma insulin levels and this was further exacerbated by supplementation with leucine (ANOVA effect of treatment p<0.001; Scheffe's posthoc test baseline – high fat p<0.001; high fat – high fat with leucine p = 0.002). **B**. High fat diet did not have a significant effect on blood glucose (ANOVA effect of treatment p = 0.054). **C**. Thus high fat diet lead to elevated HOMA-IR and this was further exacerbated by leucine supplementation (ANOVA effect of treatment p<0.001; Scheffe's posthoc test baseline – high fat p = 0.010; high fat – high fat with leucine p = 0.007). **D**. Compared to baseline levels, HF diet lead to increased FFA in both genotypes and this increase was completely reversed by leucine (ANOVA effect of treatment p<0.001; Scheffe's posthoc test baseline – high fat p = 0.029; high fat – high fat with leucine p = 0.045). Data are expressed as means ± SEM; n = 20/group at baseline and 8–11/group after high fat ± leucine.

We focused on analysis of sera from males, since most of the effects of the *SLC6A15* deletion were observed in males. At baseline, there were no differences between the WT and KO animals in serum levels of FFA, triglycerides, insulin, glucose or in HOMA-IR (Table 1; t-test p = 0.766, 0.220, 0.691, 0.487, and 0.588, respectively). Nine weeks on high fat diets resulted in significant changes in most of these parameters for mice of all genotypes (Table 1). Compared to baseline, high fat diet increased insulin resistance, as reflected by elevated HOMA-IR values. This increased resistance was further exacerbated when the high fat diet was supplemented with leucine (Figure 7C, ANOVA effect of treatment p<0.001). The increased insulin resistance was characterized by increased insulin secretion (Figure 7A; ANOVA effect of diet p<0.001) needed to maintain glucose levels similar to baseline levels (Figure 7B; ANOVA effect of diet p = 0.054). Contrary to its effects on HOMA-IR, leucine had a positive effect on plasma levels of free fatty acids. Compared to baseline levels, high fat diet increased free fatty acid levels in both genotypes. This increase was completely reversed by supplementation with leucine, which decreased free fatty acid levels below baseline values (Figure 7D; ANOVA effect of diet p<0.001).

**Table 1:** Effect of diet and genotype on serum parameters.

	+/+			−/−			Effect of diet	Effect of GT	Diet * GT
	BL	HF	LeuHF	BL	HF	LeuHF			
**FFA** (SEM)	0.374 (0.028)	0.430 (0.045)	0.254 (0.032	0.360 (0.038)	0.523 (0.069	0.269 (0.046)	<0.001	0.371	0.416
**TG** (SEM)	67.1 (3.84)	86.2 (8.33)	77.9 (10.2)	73.9 (4.04)	77.1 (6.55)	102.1 (14.6)	0.016	0.218	0.112
**Glucose** (SEM)	204 (7.95)	193 (9.67)	229 (18.9)	212 (8.06)	183 (12.0)	202 (12.7)	0.054	0.279	0.243
**Insulin** (SEM)	0.527 (0.046)	2.29 (0.618)	3.42 (0.397)	0.503 (0.040)	1.67 (0.495)	2.84 (0.275)	<0.001	0.148	0.460
**HOMA-IR** (SEM)	0.268 (0.020)	1.182 (0.367)	1.589 (0.184)	0.297 (0.052)	0.697 (0.204)	1.455 (0.203)	<0.001	0.140	0.434

BL = baseline, HF = high fat diet, LeuHF = High fat diet supplemented with leucine. The last three columns contain ANOVA p values. FFA = free fatty acids, TG = triglycerides (mg/dl), Glucose (mg/dl), insulin (ng/ml).

### Humans

We focused on associations with three common SNPs in the 5′ end of the SLC6A15 gene, including rs17183577, the A/T SNP which substitutes asparagine for valine in the alternative SLC6A15 transcript.

#### Swedish samples

**Figure 8:**
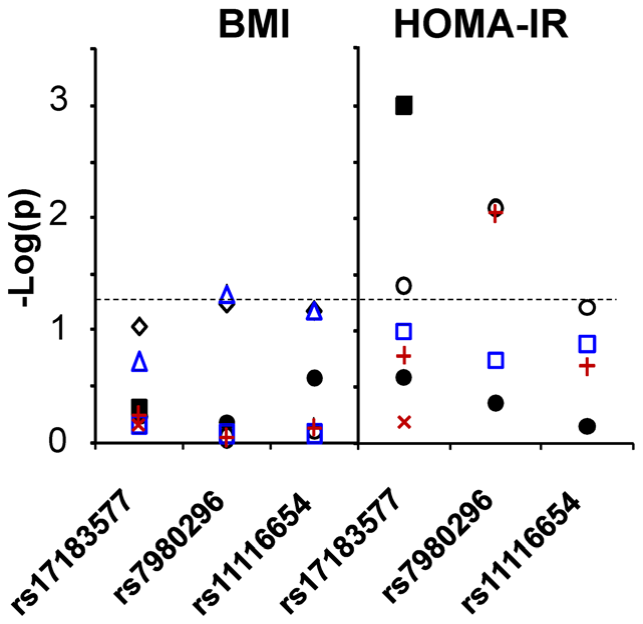
Association of the markers in SLC6A15 with body mass and insulin resistance in human samples. **Left panel:** There were trends toward association of the SLC6A15 SNPs with BMI among the normal-weight Swedish subjects, (linear regression rs17183577 p = 0.094 (all), p = 0.188 (boys), p = 0.359 (girls); rs7980296 p = 0.059 (all), p = 0.047 (boys), p = 0.433 (girls); and rs11116654 p = 0.068 (all), p = 0.066 (boys), p = 0.491 (girls). **Right panel**: There was a significant association between HOMA-IR in all the obese Swedish subjects and two of the SLC6A15 SNPs (linear regression rs17183577 p = 0.04; rs7980296 p = 0.008) and a trend in the third SNP (rs11116654 p = 0.063). rs17183577 also showed significant association with HOMA-IR in the NIH boys (linear regression p = 0.001) but not NIH girls (linear regression p = 0.73). Dashed line corresponds to p = 0.05. Symbols: ○ obese Swedish all, □ obese Swedish boys, ◊ normal Swedish all, Δ normal Swedish boys, • NIH all, ▪ NIH boys, + obese Swedish girls, × NIH girls.

Minor allele frequencies for these SNPs were 0.15–0.37. None of these SNPs revealed deviation from Hardy Weinberg equilibrium (*data not shown*). In the obese samples, HOMA-IR provided significant overall associations for two of the three SLC6A15 SNPs (Figure 8; rs17183577 p = 0.04, β = 0.13; rs7980296 p = 0.008, β = 0.134) when adjusted for age, sex and BMI. There was a trend toward association for the third SNP, rs11116654 (p = 0.063, β = 0.107). rs7980296 also displayed significant association with BMI among the normal weight boys (Figure 7; p = 0.047).

#### NIH research volunteer samples

Minor allele frequencies were 0.21–0.36 in the European-American research volunteers. Among the African-American subjects, the “minor” allele frequencies were 0.05, 0.67, and 0.30 for rs17183577, rs7980296, and rs1116654, respectively. As found in the Swedish overweight sample, there were no statistically significant associations with BMI. Linear regression of the data from 277 boys in the NIH cohort, however, provided a striking association between rs17183577 genotypes and HOMA-IR (Figure 7; p = 0.001, β = −0.137) when adjusted for age, race, and BMI-Z. The associations with HOMA-IR were statistically significant in males from the NIH sample who self-reported European ancestry (p = 0.004, β = −0.151; n = 160), but not in the smaller cohort of African-American subjects (p = 0.162, β = −0.093; n = 100). A similar association was not identified among girls (p = 0.73).

## Discussion

The most robust consequence of SLC6A15 deletion was the sex-specific interaction of diet with genotype. With leucine-supplemented water, KO males consumed more calories than their WT littermates in three different circumstances: 1) With leucine supplementation. Leucine suppressed consumption of standard laboratory chow in WT male mice but did not in SLC6A15 KO males (Figure 1). 2) During two-bottle leucine preference testing. KO males consumed more chow than their WT littermates (Figure 2). 3) On high-fat diet. Leucine supplementation resulted in more calorie intake by KO males compared to the WT controls (Figure 5). Despite higher calorie intake with supplemental leucine, KO males gained less weight than WT males. After extended exposure to leucine, this difference reached statistical significance. Analysis of abdominal fat pads suggested that the lower weight gain found in SLC6A15 KO males could be partially explained, at least, by their lower adiposity.

The observed failure of leucine to regulate appetite in SLC6A15 KO males seemed to be specific. Lysine, a ketogenic essential amino acid like leucine but, unlike leucine, a poor substrate for SLC6A15 [26], [27], suppressed appetite equivalently in SLC6A15 WT and in KO mice. Although the SLC6A15 mice did not show as strong a preference for this amino acid over water as they did for leucine in the two-bottle experiment, they consumed lysine in similar amounts as they consumed leucine (t-test p = 0.684 for WT and 0.060 for KO males).

SLC6A15 knockout females displayed differences from males: whether with or without supplemental leucine, KO females consumed the same amount of calories as their WT siblings. Contrary to KO males, SLC6A15 KO females gained more weight on leucine-supplemented high fat diet but unlike males, the differences in their weight gain were not accompanied by significant differences in the amounts of abdominal fat. This was probably due to the actions of estrogen, which regulates body fat distribution and prevents accumulation of visceral fat in favor of the subcutaneous fat [44]. Excess leucine, combined with deficiency in its transport and/or compartmentalization, thus appears to exert opposite effects in females than in males. It leads to increased adiposity in the former and decreased adiposity in the latter without affecting sex-specific distributions of fat reserves. These opposite effects may stem from the interactions between leucine transport/compartmentalization (or the lack of thereof) and sex hormones, such as estrogen, which has been shown to exert a major influence on energy homeostasis [45]. Future studies of double SLC6A15/αER KO or ovariectomized SLC6A15 KOs might help to clarify the role of sex hormones in the gender-specific effects observed in this study. These data show that while SLC6A15 deletion by itself had no effect on energy efficiency of food in either sex, when accompanied by increased leucine intake, it resulted in significant changes in efficiencies in both males and females, although in the opposite directions. Differences in feed energy efficiencies point to likely changes in energy expenditure either due to changes in locomotor activity or possibly in metabolic rates. Future studies involving metabolic cages and activity monitors will shed more light at the mechanisms involved.

Although some role of SLC6A15-expressing peripheral tissues, such as muscle, in the effects reported here cannot be excluded, increased expression of the orexigenic peptide NPY in hypothalamus of the SLC6A15 knockout mice supports, at least to some degree their brain origin. SLC6A15 expression in other brain regions such as amygdala or hippocampus might conceivably influence food-related behaviors as well. As hippocampal expression of SLC6A15 has been linked to depression in humans [46], the differences in feeding behavior of the SLC6A15 mice might have been a manifestation of their resistance to depression-induced anhedonia caused by isolation stress. However, without leucine supplementation, singly housed SLC6A15 mice consumed as much of the highly palatable high-fat chow as the control animals. Since *SLC6A15* deletion results in reduced sodium-dependent leucine uptake into brain synaptosomes [33], it is tempting to relate changes in transport and/or compartmentalization of leucine to hypothalamic mTOR signaling and its downstream appetite-regulating effects. For example, brain mTOR regulation *via* changes in activity of voltage gated calcium channels can be readily demonstrated [47], [48]. It is thus conceivable that electrogenic effects of sodium-dependent transport by SLC6A15 after diet-induced transients in leucine levels could contribute to altered mTOR regulation that follows leucine ingestion. This mechanism would be consistent with the prompt increments in arcuate hypothalamic neuronal firing rates that can be observed when leucine is applied [22].

We identified an association of markers in *SLC6A15* with obesity related phenotypes in each of two independent samples of boys. There were significant associations with HOMA-IR, when corrected for appropriate covariates, in the obese Swedish boys and in the predominantly overweight NIH sample. Other lines of evidence that support the role of *SLC6A15* in obesity related phenotypes include a prior linkage study for individual differences in carbohydrate and protein consumption that identified a 12q23.3 locus near the *SLC6A15* [12] and the associations between obesity and haplotypes in a related neutral amino acid transporter, SLC6A14 [31],[32]. By contrast, there was no strong association with BMI *per se*, though there were trends toward significance in the normal-weight Swedish samples. Since the NIH sample was enriched for overweight participants (BMI ≥95th percentile), any moderate effects of SLC6A15 polymorphisms might have been masked by other, more severe metabolic dysfunctions.

In our study, leucine supplementation did not reduce high-fat diet-induced weight gain in WT C57Bl/6 males. This is in agreement with results of several previous studies [36], [41], [49] but not others [37], [43], [50]. Effects of leucine on insulin signaling remain uncertain. Leucine activates mTOR pathways that can inhibit early steps in insulin signaling pathways, leading to insulin resistance [6], [51]. Our observations of increased insulin levels and HOMA-IR in leucine-supplemented animals after random feeding are consistent with such a biochemical mechanism. Others observed decreased [37] or unchanged [43] insulin levels and/or lower [41] or unchanged [36] glucose levels in leucine-supplemented animals which have been allowed to feed at random. In studies that measured glucose levels after glucose challenge in fasting animals, leucine-fed animals displayed either improved [41] or unaffected [43] control of postprandial rises in blood glucose. Insulin challenge showed that leucine supplementation may increase [37] or have no effect [41]on insulin sensitivity. These differences might be explained by differences in composition of the diets (fat and protein content), method of leucine supplementation (drinking water *vs* pellet), genetic and environmental backgrounds of animals or study duration, although all these parameters were similar in the studies by Nairizi and Zhang [36], [37] that produced divergent results. To ascertain leucine's role in high fat diet-induced obesity and metabolism deregulation, it may be necessary to investigate other origins of the variable responses to high fat diets, such as housing conditions that may affect activity and/or stress levels, or epigenetic factors [52].

The current data provide strong support for the idea that transport and/or compartmentalization of leucine, and perhaps other BCAA, is an important determinant of leucine-induced suppression of eating. The parallels between mouse and human data presented here are not complete. Nevertheless, it is striking that the sex specificity found in the human association corresponds to results from mice.

There are parallels between several of the mouse and human results obtained here that extend beyond association with diet/obesity related phenotypes. When fed a standard laboratory diet, *SLC6A15* KO mice are able to maintain the same weight as their WT littermates [33], in line with the weak association between the SNPs in SLC6A15 and BMI in humans. The *SLC6A15* association with HOMA-IR was only observed in overweight human samples, matching the lack of significant differences between the WT and *SLC6A15* KO mice in HOMA-IR measures when even the mice exposed to high fat diets were only 10% heavier than those exposed to low fat diets. In future studies it will be important to define more of the steps that link leucine compartmentalization to regulation of calorie intake by leucine and to the likely concomitant effects on insulin resistance and BMI phenotypes in humans.

There are caveats to consider in interpreting our current results. While deletion of a gene in laboratory animals may help elucidate its physiological roles, null mutations in humans are quite rare. Parallels between the animal and human data have thus to be drawn with great care. Current data come from leucine supplementation in drinking water, as has been previously studied [36], [37], [41]. Since SLC6A15 KO mice of both sexes consumed more leucine than the WT mice, leucine was likely to exert stronger metabolic influences in the knockouts than wildtype littermates. We did observe a significant negative correlation between leucine consumption and energy efficiency in males (r = −0.748, p<0.001) although not in females (r = 0.021, p = 0.922). Adding leucine to the chow as a primary manipulation might have also provided a similar metabolic influence, since the KO males consumed more of the chow then the WT males.

These observations in mice and humans support a novel, gender-selective role for brain amino acid compartmentalization mediated by *SLC6A15* in diet and obesity-associated phenotypes. This work provides one of the first reports of gender-specific effects of leucine supplementation on food intake and energy homeostasis, underscoring the importance of studying mice of both sexes. These results add to understanding of the complex mechanisms that regulate diet and obesity. They point toward the power of mouse/human translational research for defining subtle gene/environment elements that play roles in the important problems raised by obesity.

## Supporting Information

Figure S1 **Effect of leucine on drinking.** Both KO males and females consumed significantly more fluids (ANOVA effect of genotype p = 0.001 in males and p<0.001 in females) and this difference was further exacerbated by leucine supplementation (ANOVA effect of leucine * genotype p = 0.002 in males and p = 0.013 in females). Scheffe's post-hoc test confirmed that leucine stimulated fluid intake in v7-3KOs only (p = 0.001 for KO males; p<0.001 for KO females) while it had no significant effect on WT animals (p = 0.922 for WT males; p = 0.120 for WT females).
(TIF)

Figure S2 **Effect of lysine on drinking.** KO males and females consumed similar amounts of fluids (ANOVA effect of genotype p = 0.097 in males and p<0.994 in females). **A**. Addition of lysine into water increased drinking in KO males only (ANOVA effect of lysine * genotype p = 0.022; Scheffe's post-hoc test p = 0.151 for WT and p<0.001 in KO). **B**. In females, lysine had a similar effect on drinking in both genotypes (ANOVA effect of lysine * genotype p = 0.514).
(TIF)

Figure S3 **Lysine preference.** In a two-bottle preference test, preference for lysine did not differ between the sexes and genotypes (ANOVA effect of sex p = 0.169, effect of genotype p = 0.256).
(TIF)

Figure S4 **Effect of high fat diet and leucine supplementation on SLC6A15 males.** **A** and **C**. On high-fat diet, leucine had no significant effect on calorie intake and weight in WT males (repeated measures ANOVA genotype p = 0.490 and 0.192, respectively). **B**. In KO males, leucine increased calorie intake (repeated measures ANOVA genotype p = 0.001) without affecting their weight (repeated measures ANOVA genotype p = 0.277).
(TIF)

Figure S5 **Effect of high fat diet and leucine supplementation on SLC6A15 females.** **A**. and **B**. On high-fat diet, leucine increased calorie intake in both WT and KO females (repeated measures ANOVA p = 0.003 and 0.002, respectively). **C** and **D** Leucine had no significant effect on weight in WT (repeated measures ANOVA p = 0.230) nor KO females (repeated measures ANOVA p = 0.290).
(TIF)

Figure S6 **Effect of high fat diet and leucine supplementation on weight gain.** Leucine supplementation affected KO males and females differently (ANOVA sex * genotype * diet p = 0.002) **A**. KO males gained significantly less weight than WT males on high-fat diet supplemented with leucine- (Scheffe's post hoc p = 0.007). **B**. KO females gained significantly more weight than WT females leucine-supplemented high fat diet (Scheffe's post hoc p = 0.007).
(TIF)
